# Irregularities of the Teeth

**Published:** 1885-04

**Authors:** Norman Kingsley

**Affiliations:** New York.


ARTICLE VI.

### IRREGULARITIES OF THE TEETH.

BY DR. NORMAN KINGSLEY, NEW YORK.

[Part of paper read before Dental Society of Scotland |

An observer with limited experience may often be misled by the appearance of teeth as they first erupt. They may seem to be growing out of the line of the arch, and that a permanent irregularity is inevitable; but many such cases need no interference. If left to themselves, they will approach regularity, and oftem assume their true position, unless the occlusion of the antagonizing teeth prevents them. But interference is demanded as soon after eruption as it becomes certain a deformity is inevitable There is then no longer justification for delay; for after that period every year increases the difficulties, both pathological and mechanical, and prejudices the stability of the change.

But all irregularities in the position of the teeth are not deformities which demand treatment; there are many departures from the normal type where neither the utility nor the beauty of these organs, nor the symmetry of surrounding features, is seriously affected by such mal-position.

The regulation of teeth involves often the wearing of fixtures which cannot be removed and cleansed as frequently as the health of the mouth demands. Their continued presence may prevoke caries of the teeth, and a prolonged treatment may seriously impair the nervous system; therefore, regulating teeth should not be undertaken without proper consideration. Such a consideration must recognize the age, sex and family type, and the relation of the features; the cause of displacement, the constitution of the teeth, and the ravages of decay; the efficiency of the teeth, the enunciation of the voice, the risk of inflammation, and the destruction of pulps; the systemic condition and endurance of the patient; the time required, and the annoyance to which he would be subjected; the means and appliances for corrcction; and, finally, the character and permanency of the changes wrought.

Regulating teeth may be undertaken under favorable circumstances at any age short of full maturity; but, all things considered, the most desirable period to begin the correction of much irregularity is when the cuspids and second molars are fully erupted. The occlusion of the teeth is an important factor in determining the permanency of the change. All attempts at correction at any age will be folly, unless the antagonizing teeth on occlusion will serve to hold them in their new positions.

Success in treatment is based on the fact that the teeth are placed on the maxillæ, surrounded by vascular, elastic, bony processes, which are easily moved or absorbed under pressure, and that reproduction of bone will follow such conditions, and make the teeth solid in their new locations. The possibilities under favorable conditions, within certain limits, are almost unbounded. Narrow jaws may be widened, protruding jaws made to recede, individual teeth moved considerable distances, and teeth elongated or shortened, or twisted in their sockets. The success of skillful efforts in this direction has been triumphant.

Some of the most marked cases have been made where the face has been deformed by a protruding or receding jaw, either upper or lower. Strictly speaking, when this occurs with the upper jaw, it is not the maxilla which is at fault, but rather the whole dental arch. Such a condition in the lower jaw is more likely to rise from a mal-articulation at the joint; but in either case; when taken at the proper age, they are quite amenable to treatment.

It is possible in correcting deformities or irregularities of the dental arch, to create a deformity of the external features. It is not always advisable to attempt to change the form and expression of a mouth were the condition is an inherited peculiarity—being a part of the family type—and where the change would involve prolonged effort, possible breaking up of a good articulation, and with the knowledge that nature will be constantly making an effort to return to the inherited type. In hereditary cases of extensive character which have been delayed till at or near maturity, we can never feel certain but that the original tendency to mal-position, so long unbroken, will re-assert itself at any time we abandon retaining fixtures.

On general principles, it is desirable to retain every sound tooth in the mouth, yet there are many cases of crowded dentition where the removal of a tooth on each side of the jaw is justifiable. The retention of every tooth in the mouth is not necessary to the efficiency of the masticating apparatus, is not required to maintain the contour of the jaw, and the loss of certain teeth produces no visible external effect. Good articulation is of more importance than the full number of teeth.—*Items of Interest.*

